# Investigation of daily patterns for smartphone keystroke dynamics based on loneliness and social isolation

**DOI:** 10.1007/s13534-023-00337-0

**Published:** 2023-11-22

**Authors:** Seokbeen Lim, Chaeyeon Kim, Baek Hwan Cho, Soo-Hee Choi, Hyeongrae Lee, Dong Pyo Jang

**Affiliations:** 1 Dept. of Biomedical Engineering, Hanyang University, Seoul, Republic of Korea; 2 Dept. of Electronic Engineering, Hanyang University, Seoul, Republic of Korea; 3 Dept. of Biomedical Informatics, CHA University School of Medicine, CHA University, Seongnam, Republic of Korea; 4 Institute of Biomedical Informatics, School of Medicine, CHA University, Seongnam, Republic of Korea; 5 Dept. of Psychiatry, Seoul National University College of Medicine and Institute of Human Behavioral Medicine, SNU-MRC, Seoul, Republic of Korea; 6 Dept. of Psychiatry, Seoul National University Hospital, Seoul, Republic of Korea

**Keywords:** Mobile keystroke dynamics, Social isolation and loneliness, Smartphone keyboard, Passive mobile sensing technology

## Abstract

This study examined the relationship between loneliness levels and daily patterns of mobile keystroke dynamics in healthy individuals. Sixty-six young healthy Koreans participated in the experiment. Over five weeks, the participants used a custom Android keyboard. We divided the participants into four groups based on their level of loneliness (*no loneliness*, *moderate loneliness*, *severe loneliness*, and *very severe loneliness*). The *very severe loneliness* group demonstrated significantly higher typing counts during sleep time than the other three groups (one-way ANOVA, *F* = 3.75, *p* < 0.05). In addition, the average cosine similarity value of weekday and weekend typing patterns in the *very severe loneliness* group was higher than that in the *no loneliness* group (Welch’s *t*-test, *t* = 2.27, *p* < 0.05). This meant that the *no loneliness* group’s weekday and weekend typing patterns varied, whereas the *very severe loneliness* group’s weekday and weekend typing patterns did not. Our results indicated that individuals with very high levels of loneliness tended to use mobile keyboards during late-night hours and did not significantly change their smartphone usage behavior between weekdays and weekends. These findings suggest that mobile keystroke dynamics have the potential to be used for the early detection of loneliness and the development of targeted interventions.

## Introduction

Social isolation and loneliness significantly affect both physical and mental health, leading to an increased risk of cardiovascular disease and even mortality [1–4]. It is crucial to detect feelings of loneliness, such as those equivalent to smoking 15 cigarettes per day before they become chronic [4].

Recently, many researchers focused on smartphone-based mobile passive data and its relationship with mental health conditions, such as mood, depression, etc. [5–10]. The advantage of mobile passive data in these studies stems from its capability to be collected unobtrusively without the user’s active involvement, coupled with the enriched temporal resolution of the data acquired [11–13]. Rosenthal et al. explored the correlation between daily mobile screen usage and depressive symptoms among college students [5]. Their findings indicated an exacerbation of depressive symptoms in users who engaged with screens for over 5.72 h daily [5]. DANG et al. collected multimodal behavioral data associated with smartphone usage, including metrics like screen on/off status, display lock/unlock actions, time spent on screen, frequently used apps, incoming and sent SMS, and acceleration [9]. Utilizing this passive data, they developed an application called FINE for the early detection and intervention of depression [9]. Fukazawa et al., on the other hand, harnessed multimodal mobile passive data, encompassing factors like ambient illuminance levels, acceleration, geolocation, app and browser usage, and email activity [10]. Their objective was to evaluate anxiety-related stress levels, and their study achieved a high predictive performance of anxiety-related stress, almost 74% of the F-score [10].

As a subset of mobile passive data, features from mobile keystroke dynamics have also been gathered to explore their associations with various mental health conditions [7, 8]. Mastoras et al. investigated the patterns of keystroke features in a group of patients with depression [8]. They developed a machine learning-based method to distinguish subjects with depressive tendencies from healthy controls based on the self-reported Patient Health Questionnaire 9 (PHQ-9) score [8]. They showed machine learning performance corresponding to an Area Under the Curve (AUC) of 0.89 and found that the probability output from the machine was significantly correlated with the PHQ-9 score [8]. Vesel and colleagues investigated the potential of daily-life keystroke metadata as a digital indicator of mood states [7]. Key input features corresponding to 14 million keys were extracted from 250 users, and nested data were also extracted for those who responded to the depression scale (Patient Health Questionnaire 8, PHQ-8) and analyzed through a metric growth curve mixed effect model [7]. The findings revealed that individuals with severe depression displayed greater typing speed variability, shorter typing sessions, and reduced typing accuracy [7]. Moreover, in terms of daily patterns, typing speed peaked around noon and showed the least variability [7]. The researchers posited that mobile keystroke data is closely related to mood states and can provide a foundation for developing digital biomarkers [7].

In the aspect of social isolation and loneliness, passive mobile sensing technology, known as context-aware computing, has been studied as a potential method for objectively measuring loneliness at an early stage [1, 4]. With the widespread use and availability of smartphones [14], it is now possible to gather new behavioral data, called digital phenotypes, from individuals using built-in sensors [15, 16]. These data can be collected passively without conscious awareness of the user, offering valuable insights into loneliness detection [11–13].

Multimodal passive data, including Bluetooth usage history, phone calls, short message service, Wi-Fi, global positioning system, screen on/off, steps, browsing patterns, and social media usage, were collected to understand behavioral patterns according to loneliness levels [11–13]. Doryab et al. confirmed that the behavioral patterns of college students vary according to their level of loneliness, based on multimodal data collected from smartphones and wearable bands, and used machine learning to classify loneliness levels [11]. Wu et al. verified the correlation between college students’ self-reported companionship types and momentary loneliness based on Bluetooth and global positioning system data and employed this information for loneliness prediction modeling [12]. Pulekar and Agu proposed a Socialoscope app that detects loneliness through user communication and interaction patterns, such as phone calls, short message service, and browsing patterns [13].

In this study, daily patterns related to varying levels of loneliness were examined using mobile keystroke dynamics in healthy individuals. Mobile keystroke dynamics have been studied in various mental health conditions [6–8]. Zulueta et al. explored the relationship between mobile keyboard features and mood disorders in patients with bipolar disorder, confirming the potential for predicting signs and symptoms of manic and depressive episodes through clinician-rated mood regulation scales [6]. They identified several significant keystroke variables for each item of keystroke metadata using a mixed-effects regression model for the depression scale and a least-squares linear regression model for the bipolar disorder scale [6]. This demonstrated that mood states in bipolar disorder might be related to keystroke-specific changes in mobile phone use [6]. However, to the best of our knowledge, few studies have explored keystroke dynamics in relation to loneliness levels. Mobile keystroke data are associated with mental health aspects such as depression and offer the advantage of identifying users’ diurnal patterns [17]. Additionally, studies have indicated that increased internet usage [18] and heightened mobile keyboard activity, such as the session count, among individuals exhibiting depressive symptoms [6], suggest that keystroke patterns might play a significant role in correlating with mental health conditions like depression. Given that the findings of previous studies have demonstrated the correlation between loneliness levels and depression, anxiety, and psychosis [19, 20], investigating daily patterns based on loneliness levels through mobile keystroke dynamic information can contribute significantly to the field of loneliness detection research.

## Methods

### Participants

We recruited participants by disseminating an Institutional Review Board of Hanyang University (HYUIRB) approved recruitment notice across various university online communities in Seoul. Additionally, we printed and posted these notices on each bulletin board throughout Hanyang University’s campus in Seoul. Individuals who wanted to participate in the experiment filled out and submitted an online participation application form including their information, such as name, age, gender, and occupation, by scanning the QR code or accessing the online application link provided on the notice. Researchers contacted the participants who used Android smartphones with QWERTY-type soft keyboards among those who submitted the online application form in advance to arrange an online meeting schedule. At the coordinated meeting date, participants received a research overview and instructions on installing the keyboard app through online meetings.

Sixty-six healthy young participants (Age range: 22.44 ± 2.03) from the Republic of Korea were enrolled in this study. The majority of them were undergraduate students, accounting for fifty-eight individuals or 88% of all participants. Additionally, there were five graduate students (8%), two employed workers (3%), and one unemployed individual (1%).

All the experimental procedures were approved by the HYUIRB (HYUIRB-202207-003-1). In addition, all participants provided written informed consent online and were given a small financial incentive to participate. The characteristics of the participants are presented in Table 1.

### Data collection

**Fig. 1:**
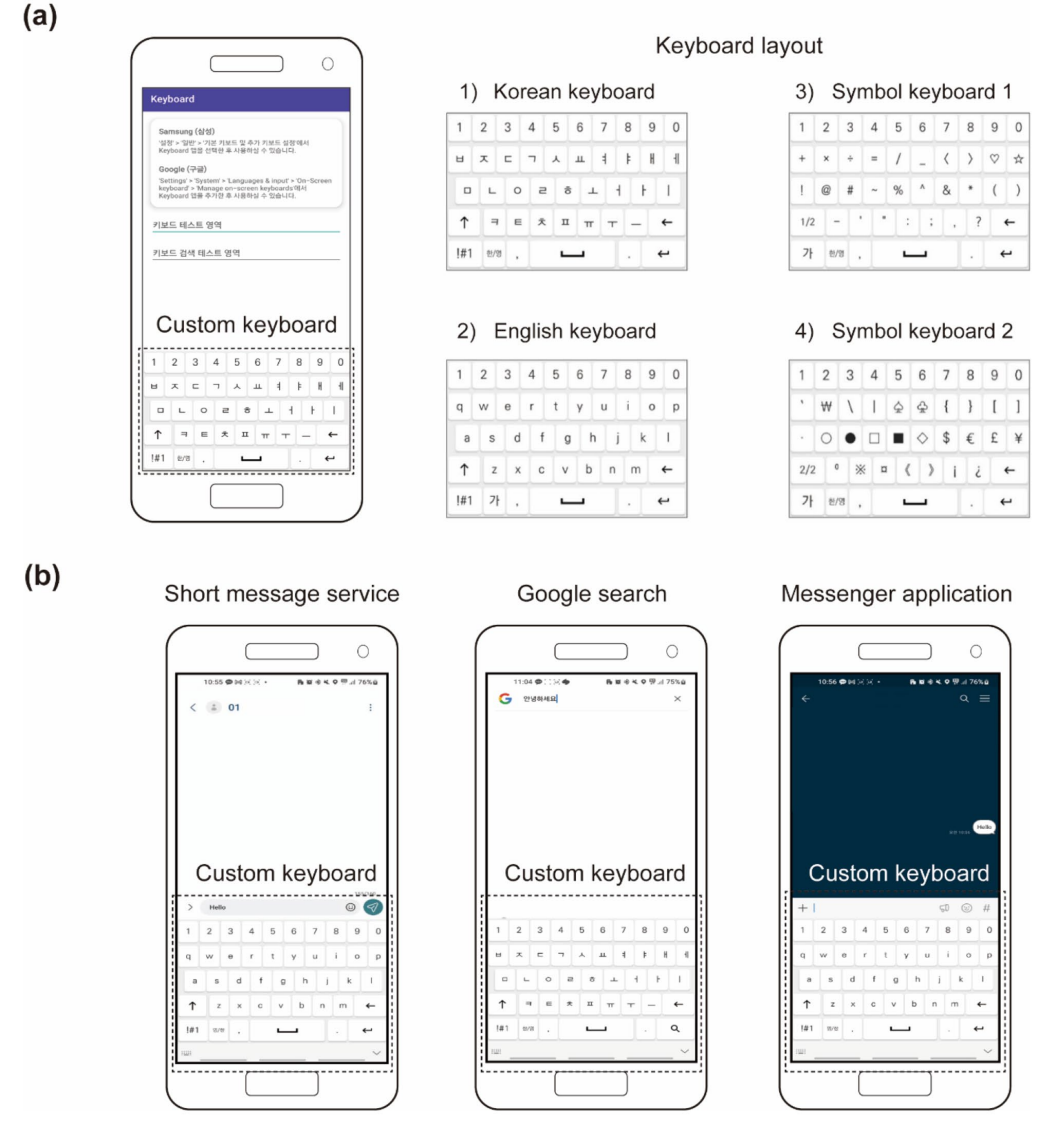
The custom keyboard was developed for Android, and divided into four layouts: Korean keyboard layout, English keyboard layout, and symbol keyboard layouts 1 and 2. Custom keyboard layout is automatically called when the user types. From left to right, short message service, google search, messenger application

QWERTY-type custom keyboard app was developed for the Android OS to record keystroke dynamics. This app uses key data anonymization protocols to protect the privacy of the user’s key input and records the timestamp of the pressing and release of each keyboard button. The data were safely uploaded daily to a web server built at Hanyang University. The custom keyboard layout consisted of Korean QWERTY, English QWERTY, and special character layouts. Detailed keyboard layouts are shown in Fig. 1a.

Participants installed the keyboard app distributed as an alpha version in the Google Play Console and interacted with their smartphones via this app in everyday life for five weeks. Upon installing our custom keyboard app, participants set it as their default keyboard. Consequently, the app operated in the smartphone’s background during everyday use. This ensured that the custom keyboard layout was automatically invoked by the device in the user’s typing environment. Examples of automatically called custom keyboard layouts when users type are shown in Fig. 1b. Our keyboard app possesses a feature that logs both when it’s set as the default keyboard and begins background operation and when it’s deselected, terminating its background activity. Using this feature, we reviewed the log data and key data after the experiment concluded to determine if any missing key data resulted from participants switching their default keyboard settings away from the custom keyboard installed during the study.

They were encouraged to complete online the translated version of the University of California, Los Angeles (UCLA) loneliness measurement scale short version (Korean version ULS-8) sent by the trained staff at the end of the five weeks [21–23].

### Processing of keystroke features

Keystroke data were collected over a span of five weeks. While most participants successfully collected their keystroke data for the entire five-week period, some lost more than a day of data due to reasons such as switching back to their other keyboard application during the experiment period. Therefore, we decided to include only those participants who had complete weekly data for at least three out of the remaining four weeks (i.e., more than 75% of one month), with the initial week excluded from the analysis as it was considered an adjustment period for the newly installed custom keyboard. During this process, seven participants were excluded from the analysis due to their failure to meet these criteria. For each participant’s extracted dataset, the typing count and typing count ratio were computed for each of the five time ranges (sleep:12:00 AM–6:00 AM; morning:6:00 AM–12:00 PM; afternoon:12:00 PM–5:00 PM; evening:5:00 PM–9:00 PM; and night:9:00 PM–12:00 AM). The typing count ratio was calculated as the ratio of the typing count for each time range to the total daily typing count. Then, the daily typing count and daily typing count ratio for each participant were extracted by averaging these values. In addition, to confirm the similarity in patterns between weekdays and weekends, we performed separate analyses of the typing count ratios for weekdays and weekends.

### Statistical analysis

The ULS-8 loneliness scale consists of 8 items with a total score of 32 points [21, 23]. Each question was assigned 4 points, with questions 7 and 8 scored inversely [21, 23]. According to the ULS-8, participants are divided into four groups based on their loneliness levels [21, 23]. The *no loneliness* group scores range from 8 to 13 points, indicating a low level of loneliness. The *moderate loneliness* group scores range from 14 to 20 points, representing moderate loneliness. The *severe loneliness* group scores range from 21 to 25 points, signifying moderate to high loneliness, and the *very severe loneliness* group scores range from 26 to 32 points, reflecting a level of loneliness above the high level.

The 59 participants included in the analysis were assigned into four groups based on their results: 18 individuals in the *no loneliness* group, 26 in the *moderate loneliness* group, 10 in the *severe loneliness* group, and 5 in the *very severe loneliness* group. The average typing count for the four groups was calculated based on the daily typing count of each participant. A one-way analysis of variance (ANOVA) was performed on the four loneliness groups for each time section using typing counts for each participant. A post-hoc analysis was conducted using Tukey’s multiple comparison test to identify significant differences between the loneliness group pairs.

Curve fitting was performed using a second-order polynomial for each participant to quantify the weekday/weekend daily typing count patterns for each group. In a quadratic polynomial, the coefficients of the quadratic term are associated with the curve shape and width. Curve fitting was performed using the polyfit function in MATLAB. One-way ANOVA of the loneliness group was conducted by extracting the quadratic coefficient from the fitted curve for each participant. In addition, post-hoc analysis was conducted using Tukey’s multiple comparison test to identify significant differences between the loneliness group pairs.

Furthermore, the cosine similarity method was employed to analyze the similarity between weekday and weekend daily typing count ratio for each group [24, 25]. Subsequently, a *t*-test was conducted between the loneliness groups using the cosine similarity value.

**Table 1:** Participant characteristics

Characteristics	Value
Total number of users, n	66
Female gender, n (%)	43 (65)
Age, mean (SD)	22.44 (2.03)
Occupation	
Undergraduate student, n (%)	58 (88)
Graduate student, n (%)	5 (8)
Employed worker, n (%)	2 (3)
Unemployed individual, n (%)	1 (1)
Number of users involved in the analysis	59
Number of keystrokes (n = 59)	
Mean (SD)	127,031 (75,774)
Questionnaire (n = 59)	
Loneliness, total mean (SD)	16.88 (5.47)
*no loneliness* group, mean (SD)	10.94 (1.51)
*moderate loneliness* group, mean (SD)	16.65 (2.15)
*severe loneliness* group, mean (SD)	22.60 (1.11)
*very severe* loneliness group, mean (SD)	28.00 (1.41)

## Results

### Daily keystroke pattern related to loneliness

**Fig. 2:**
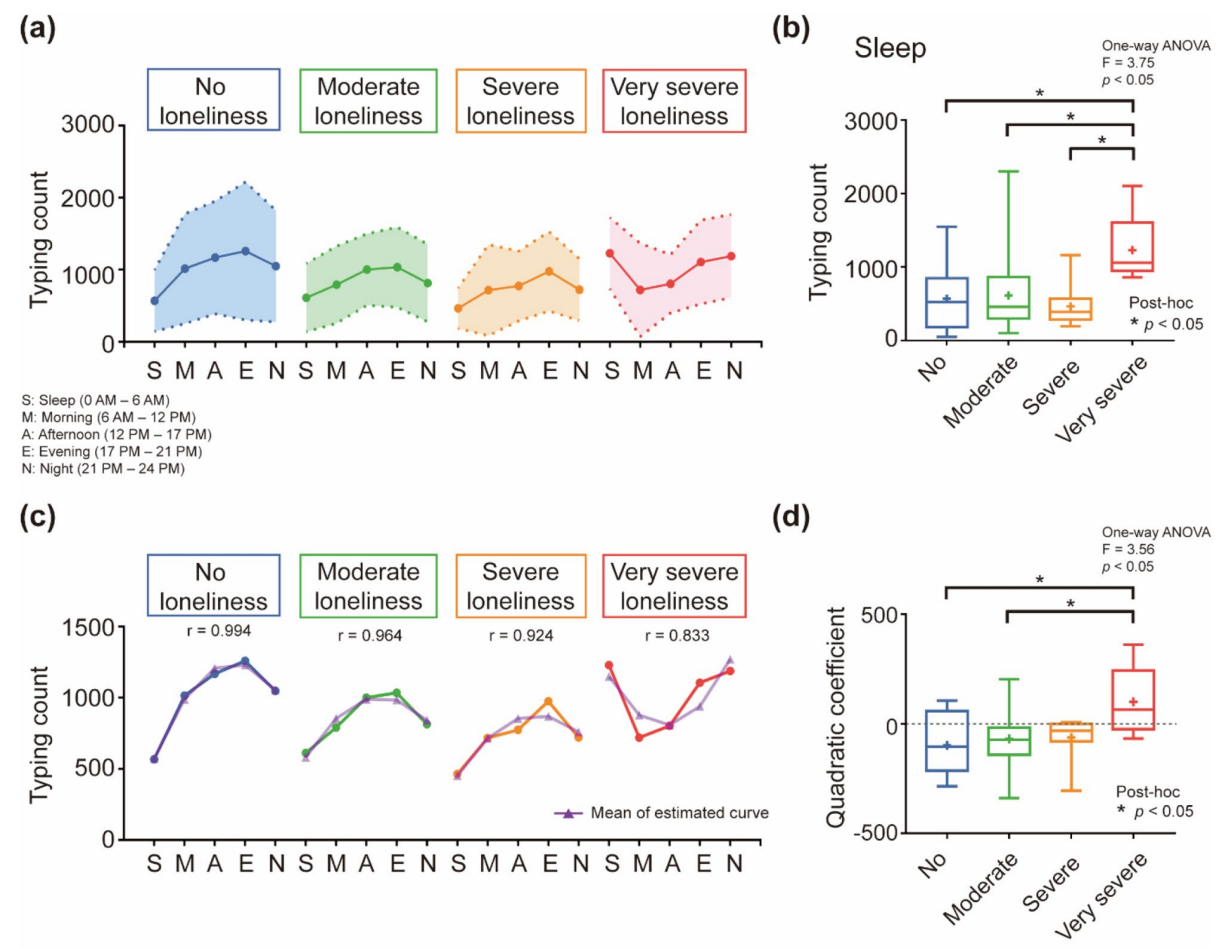
Daily typing count for each loneliness group. (**a**) The average typing count is depicted, with ranges representing standard deviations. Time was divided into five sections: sleep, morning, afternoon, evening, and night. The average typing count for the *no loneliness*, *moderate loneliness*, and *severe loneliness* groups increased toward evening time. However, the *very severe loneliness* group exhibited a high mean typing count during late-night hours. (**b**) One-way ANOVA results of the typing count during sleep time. The average typing count of the *very severe loneliness* group was higher than those of the other groups during sleep time. The cross shape in the box plot denotes the mean, and the long line signifies the median. (**c**) Second-order polynomial curve fitting to mean typing count. Both the fitted curve of quadratic coefficient and mean typing count were overlapped. “r” denotes the correlation coefficient between the original and estimated mean typing count. (d) One-way ANOVA of quadratic coefficients of the fitted curve for each participant. The *very severe loneliness* group with a positive mean of the quadratic coefficient showed a significant difference from the *no loneliness* and *moderate loneliness* groups with a negative mean of the quadratic coefficient. The cross shape in the box plot denotes the mean, and the long line signifies the median

We categorized each participant into one of the four groups based on their level of loneliness and calculated the average typing count by dividing the day into five distinct periods. For the *no loneliness*, *moderate loneliness*, and *severe loneliness* groups, the average typing count increased in the evening, as shown in Fig. 2a. However, unlike the other groups, the *very severe loneliness* group exhibited a high average typing count during the late-night hours (i.e., night and sleep).

ANOVA of the four loneliness groups for each period revealed a significant difference only in sleep time (F = 3.75, *p* < 0.05), as shown in Fig. 2b. The average typing count of the *very severe loneliness* group was higher than those of the other groups during sleep time.

Daily typing counts were also extracted as quantitative indicators using quadratic curve fitting. Curve fitting was performed on the average typing count for each participant, and the estimated curves were averaged for each loneliness group. As shown in Fig. 2c, the estimated average typing count showed a strong correlation, with the correlation coefficient ranging from 0.83 to 0.99.

An ANOVA was performed on the four loneliness groups by extracting the quadratic coefficient of the fitted curve for each participant, as shown in Fig. 2d. The *very severe loneliness* group, with a positive mean quadratic coefficient, showed a significant difference from the *no loneliness* and *moderate loneliness* groups, with a negative mean quadratic coefficient (*F* = 3.56, *p* < 0.05).

### Weekday and weekend typing pattern analysis

**Fig. 3:**
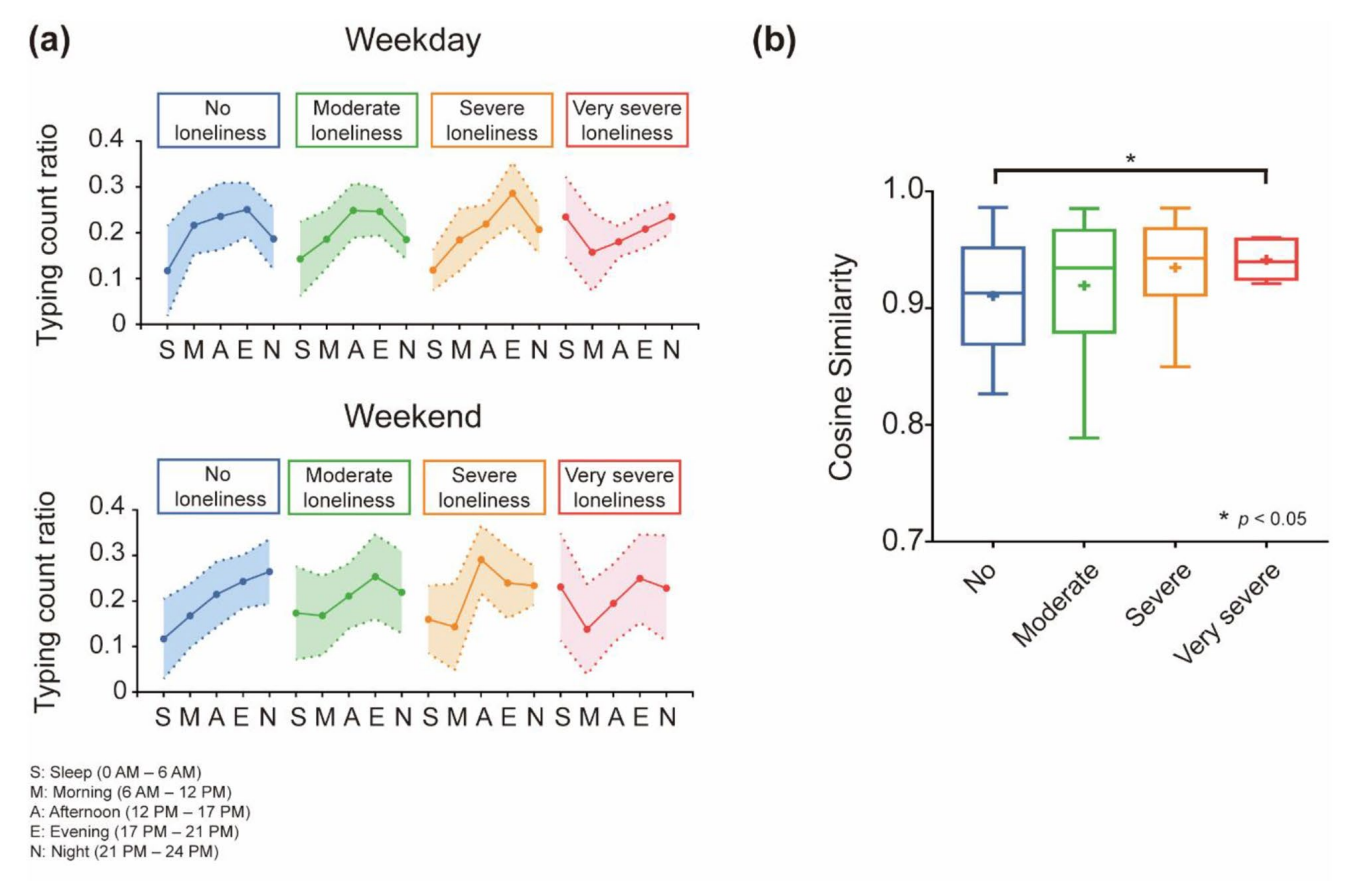
Comparison of mean typing count ratio between weekdays and weekends. (**a**) Mean typing count ratio of weekdays and weekends for each loneliness group. The average typing count is depicted, with ranges representing standard deviations. Time was divided into five sections: sleep, morning, afternoon, evening, and night. The *very severe loneliness* group had the highest average typing count ratio in the late-night hours on both weekdays and weekends and the lowest in the morning. (**b**) Cosine similarity between the weekday and the weekend typing count ratio for each group. The similarity of the typing patterns on the weekday and weekend of *the very severe loneliness* group was significantly higher than the similarity of the *no loneliness* group

The typing count ratios for weekdays and weekends were extracted for each participant and averaged for the loneliness group, as shown in Fig. 3a. The *very severe loneliness* group had the highest average typing count ratio in the late-night hours on both weekdays and weekends, and the lowest in the morning. The cosine similarity value for the weekday/weekend typing count ratio demonstrated that the similarity of the typing patterns on the weekday and weekend of the *very severe loneliness* group was significantly higher than the similarity of the *no loneliness* group (Welch’s *t*-test, *t* = 2.27, *p* < 0.05).

## Discussion

In this study, we investigated the relationship between loneliness levels and daily patterns of mobile keystroke dynamics in healthy individuals. Our findings revealed distinct diurnal patterns of smartphone typing behavior in relation to loneliness levels, with noticeable differences between weekdays and weekends. To the best of our knowledge, this is the first study to explore the association between loneliness and mobile keystroke dynamics.

The results demonstrated that the *no loneliness*, *moderate loneliness*, and *severe loneliness* groups exhibited an average typing count increase toward the evening time, while the *very severe loneliness* group showed a high average typing count during late-night hours. These findings suggest that individuals with very severe levels of loneliness engage in different daily typing behaviors than individuals with lower levels of loneliness. ANOVA further confirmed significant differences in typing counts between the *very severe loneliness* group and the other groups during sleep time. This observation could indicate that individuals with very severe loneliness may be more prone to using their smartphones during late-night hours, which might lead to potentially exacerbated feelings of loneliness and thus negatively impact their mental health.

Our results revealed that the daily typing count of the *very severe loneliness* group exhibited a high count during the night and sleep time, suggesting that they may belong to the evening chronotype. Evening chronotypes, often referred to as night owls, exhibit peak productivity and high energy levels in the evening [26]. These individuals are psychologically vulnerable and experience significantly worse outcomes in terms of depression, anxiety, sleep disorders, and social support than other chronotypes [26]. Chang et al. reported an association between social loneliness trajectories during adolescence and evening chronotypes in late adolescence, emphasizing the need for early interventions targeting psychological factors to prevent social loneliness in evening chronotypes [27]. As previously reported [26, 27], this could be a psychologically vulnerable state, potentially leading to social isolation and heightened loneliness. Therefore, we believe that daily keystroke patterns could reflect an individual’s chronotype, and preventive solutions could be provided after identifying individual smartphone usage patterns.

Another noteworthy finding of this study is the difference in patterns between weekdays and weekends. The *no loneliness* group had a progressively higher average typing count ratio from the morning to evening on weekdays. However, on weekends, the average typing count ratio was lower in the morning and highest at night. The *very severe loneliness* group had the highest average typing count ratio in the late-night hours on both weekdays and weekends, and the lowest in the morning. In addition, the cosine similarity value for the weekday/weekend typing count ratio showed that the *very severe loneliness* group had patterns of typing on weekends and weekdays with significantly higher similarity than the *no loneliness* group. This observation suggests that individuals with very severe loneliness may not experience significant changes in their smartphone usage behavior between weekdays and weekends. In this case, individuals experiencing social isolation and loneliness might maintain irregular routines in daily life without distinguishing between weekdays and weekends. Conversely, we considered that those who were mainly active during nighttime hours, regardless of whether they were on weekdays or weekends, could feel socially isolated or lonely because of their lifestyle patterns.

Furthermore, the daily typing count pattern exhibited a gradual decrease in the average count from the *no loneliness* to the *severe loneliness* group, as shown in Fig. 2c. In contrast, the *very severe loneliness* group demonstrated a similar average typing count to the *no loneliness* and *moderate loneliness* groups. Studies have found that as loneliness levels increased, the behavioral range declined, and with higher levels of depression, which correlate with loneliness levels, texting frequency and activity, such as steps, decrease [11, 28]. Narziev et al. reported that higher depression scores are associated with reduced physical and social activities. However, the group with the highest depression score displayed activity levels similar to those of the control group, possibly because of the psychological phenomenon of overcompensation [29]. Our results also indicate that the *very severe loneliness* group had an average typing count similar to that of the *no loneliness* and *moderate loneliness* groups, which could be interpreted as a tendency to use smartphones more frequently for the psychological phenomenon of overcompensation due to heightened feelings of loneliness.

The findings of this study have significant implications for the early detection of loneliness and the development of targeted interventions. By monitoring an individual’s mobile keystroke dynamics, it may be possible to identify those at risk of experiencing high levels of loneliness and provide timely support or interventions to mitigate the negative consequences of loneliness on mental health. Because, however, the participants in this study were primarily in their 20s, additional research is required to ascertain whether the study’s results are applicable to various age groups. Loneliness is not only solely a problem for young individuals but also it may pose a more severe issue for older people [30]. Thus, there is a need for further investigation to validate these findings in larger and more diverse populations, as well as to explore additional features of smartphone usage that may be associated with loneliness levels.
